# Simultaneous Determination of Crypto-Chlorogenic Acid, Isoquercetin, and Astragalin Contents in *Moringa oleifera* Leaf Extracts by TLC-Densitometric Method

**DOI:** 10.1155/2013/917609

**Published:** 2013-02-25

**Authors:** Boonyadist Vongsak, Pongtip Sithisarn, Wandee Gritsanapan

**Affiliations:** Department of Pharmacognosy, Faculty of Pharmacy, Mahidol University, 447 Sri-Ayudthaya Road, Ratchathevi, Bangkok 10400, Thailand

## Abstract

*Moringa oleifera* Lamarck (Moringaceae) is used as a multipurpose medicinal plant for the treatment of various diseases. Isoquercetin, astragalin, and crypto-chlorogenic acid have been previously found to be major active components in the leaves of this plant. In this study, a thin-layer-chromatography (TLC-)densitometric method was developed and validated for simultaneous quantification of these major components in the 70% ethanolic extracts of *M. oleifera* leaves collected from 12 locations. The average amounts of crypto-chlorogenic acid, isoquercetin, and astragalin were found to be 0.0473, 0.0427, and 0.0534% dry weight, respectively. The method was validated for linearity, precision, accuracy, limit of detection, limit of quantitation, and robustness. The linearity was obtained in the range of 100–500 ng/spot with a correlation coefficient (*r*) over 0.9961. Intraday and interday precisions demonstrated relative standard deviations of less than 5%. The accuracy of the method was confirmed by determining the recovery. The average recoveries of each component from the extracts were in the range of 98.28 to 99.65%. Additionally, the leaves from Chiang Mai province contained the highest amounts of all active components. The proposed TLC-densitometric method was simple, accurate, precise, and cost-effective for routine quality controlling of *M. oleifera* leaf extracts.

## 1. Introduction

*Moringa oleifera* Lam. (Moringaceae) is a small to medium evergreen tree widely distributed in Asia, Africa, and America. The plant is not only well known for high nutritional contents but also recognized for its therapeutic values [1]. The leaves of *M. oleifera* have been indigenously used for various medicinal purposes such as treating bronchitis, controlling glucose level, and reducing glandular swelling [1, 2]. Numerous pharmacological investigations of* M. oleifera* leaves have been reported on anti-inflammation, anti-infection, antidiabetic, antioxidant, and antihyperlipidemic activities [3–7]. Recently, isoquercetin, astragalin, and crypto-chlorogenic acid were reported to be major active components in *M. oleifera* leaves [8]. Isoquercetin is a powerful natural antioxidant which possesses several potential therapeutic effects including antiasthma and antihypertension [9–11]. Astragalin is also reported as a natural antioxidant agent exhibiting some biological properties such as attenuation of inflammation, inhibition of dermatitis, and cellular protective effect [12–14]. Chlorogenic acid and its isomers are esters of quinic and caffeic acids that have abilities to inhibit oxidation and also promote various pharmacological activities such as antiobesity, reduction of plasma and liver lipids, and inhibition of acute lung injury [15–18].

Standardization of herbal extracts is essential to ensure their quality and biological activities. Some analytical techniques including high performance liquid chromatography (HPLC) and liquid chromatography-mass spectrometry (LC-MS) were previously developed for the quantitative analysis of the *M. oleifera* leaf extract [8, 19]. However, a simple, rapid, and inexpensive method for routine analysis of major active constituents in the plant is still preferred. Thin-layer-chromatography (TLC-)densitometry is one of the suitable methods popularly used for quality control of botanical extracts because of its fast data acquisition, simplicity, and reliability [20, 21]. Moreover, there has been no report on simultaneous quantification of isoquercetin, astragalin and crypto-chlorogenic acid in *M. oleifera* leaf extracts by this method before. Thus, the objectives of this work were to develop and validate a TLC-densitometric method for quantitative analysis of these principle constituents in the extracts of *M. oleifera* leaves collected from different locations in Thailand and to find good sources of this plant's raw material for pharmaceutical and nutraceutical development.

## 2. Materials and Methods

### 2.1. General

*M. oleifera* leaves were collected during January to March 2011 from 12 different provinces in all parts of Thailand, those are, Chiang Mai, Lampang, Surin, Udonthani, Sa Kaeo, Chonburi, Ang Thong, Saraburi, Kanchanaburi, Phetchaburi, Phang Nga, and Phuket. The samples were identified by Dr. W. Gritsanapan; the voucher specimens (BVMO11001-BVMO11012) were deposited at Department of Pharmacognosy, Faculty of Pharmacy, Mahidol University, Thailand. The leaves were dried in a hot air oven at 60°C for 24 hours. The dried samples were ground and passed through a sieve (20 mesh), and stored at room temperature (28 ± 2°C) protected from light till taken. All reagents and solvents were of analytical grade. TLC was carried out on precoated silica gel GF_254_ sheets (Merck, Darmstadt, Germany). Pure compounds, isoquercetin, astragalin, and crypto-chlorogenic acid, isolated and identified from our previous work [8], were used as standard compounds.

### 2.2. Apparatus

A Linomat 5 automatic sample spotter (CAMAG, Muttenz, Switzerland) and a 100 *µ*L syringe (Hamilton, Bonaduz, Switzerland) were used. A glass twin-trough chamber (20 × 10 × 4 cm, CAMAG), TLC scanner 3 linked to winCATS software (CAMAG), and TLC plates of 20 × 10 cm with 0.2 mm layer thickness, precoated with silica gel 60 GF_254_ (Cat. No. 1.05554.0001, Merck), were used in this experiment.

### 2.3. Preparation of Standard and Sample Solutions

Stock solutions of isoquercetin, astragalin, and crypto-chlorogenic acid were prepared by dissolving each standard compound in 50% methanol in a volumetric flask at a concentration of 1,000 *µ*g/mL. The stock solution was diluted with 50% methanol to provide five working standard solutions (concentrations of 20, 40, 60, 80, and 100 *µ*g/mL).

Each sample of *M. oleifera* powder leaves was accurately weighed (5.0 g) and extracted by maceration with 70% ethanol (1 : 20, w/v), which is the most suitable extraction method [22], for 72 hours at room temperature with occasional shaking. The extraction process was repeated for 5 times to provide exhaustive extraction. The extracts were combined, filtered, and dried under vacuum in a rotary evaporator. The dried extract was adjusted with 50% methanol to a 10 mL volume in a volumetric flask. The solution was filtered using a 0.45 mm nylon membrane filter before application onto the TLC plate. Each sample was prepared and analyzed in triplicate.

### 2.4. Validation of the Method

The analytical method was validated for linearity, precision, accuracy, limit of detection (LOD), limit of quantitation (LOQ), and robustness according to International Conference on Harmonization (ICH) guidelines [23].

#### 2.4.1. Linearity

Linearity was determined using the standard solution in 50% methanol. Five microliters of five concentrations (20, 40, 60, 80, and 100 *µ*g/mL) of each reference standard were individually prepared and spotted on the TLC plate to obtain the calibration range of 100–500 ng/spot. The calibration graphs were acquired by plotting the peak area versus the concentration of the standard solutions.

#### 2.4.2. Precision

The precision was determined by analyzing 200, 300, and 400 ng/spot of each standard solution after the application by the proposed method onto a TLC plate on the same day for intraday precision and on three consecutive days for interday precision. The precision was expressed as percent relative standard deviation (RSD).

#### 2.4.3. Accuracy

The accuracy of the analyzing method was calculated by performing recovery studies of three levels of each standard (isoquercetin, astragalin, and crypto-chlorogenic acid) added to the sample solution. The solutions were applied onto a TLC plate and analyzed by the proposed method. Three analyses were performed for each concentration level of the standards. The average recoveries were calculated as recovery (%) = 100 × (amount found – original amount)/amount added.

#### 2.4.4. LOD and LOQ

LOD and LOQ were determined by preparing five different levels (20, 40, 60, 80, and 100 *µ*g/mL) of each standard stock solution and used accordingly. Blank methanol was also spotted three times following the same method and the signal-to-noise ratio was determined. The LOD was considered as 3 : 1 and the LOQ as 10 : 1. The LOD and LOQ were experimentally verified by diluting the known concentration of standards until the average responses were approximately 3 or 10 times of the standard deviation of the response, for three replicate determinations of LOD and LOQ, respectively.

#### 2.4.5. Robustness

The robustness of the method was evaluated by introducing little changes in certain chromatographic parameters at each standard concentration level of 200 ng/spot. The ratio of mobile phase composition was changed slightly as 34 : 3.5 : 1.5 : 7.30 : 3 : 1.5 : 5 and 32 : 3.5 : 1.5 : 6, v/v/v/v, for ethyl acetate : formic acid : acetic acid : water, respectively. The length of the chromatogram on TLC plate was varied, 70 mm, 80 mm, and 90 mm. The periods of time between spotting the standards onto the TLC plate and developing the plate (5, 15, and 30 minute), duration of TLC tank saturation (30, 60, and 90 minute) and duration between TLC plate development and scanning (5, 30, and 60 minute) were varied. The RSD values of the peak areas of standards were calculated for all variations.

### 2.5. Chromatographic Conditions

The TLC plates were pre-washed with methanol and activated at 105–110°C for 15 minutes before use. The samples were spotted as 7 mm bands wide with a 100 *µ*L Hamilton syringe at 10 mm from the bottom edge of TLC plate using a CAMAG Linomat 5 automatic sample spotter and nitrogen gas with a constant rate of 80 nL/s. Each sample solution was applied in triplicate. The mobile phase consisted of ethyl acetate : formic acid : acetic acid : water, 34 : 3.5 : 1.5 : 7, v/v/v/v. Linear ascending development was performed in a twin through glass chamber presaturated with the mobile phase for 60 minutes at room temperature. The length of the chromatogram run was 80 mm and the developing time was around 25 minutes. Densitometric scanning was performed using a CAMAG TLC 3 scanner in the reflectance-absorbance mode at 340 nm controlled by winCATs software. The slit dimension was 6.00 × 0.45 mm with a scanning speed of 20 mm/s. Five-point calibration was performed for each analysis by the proposed method. The amounts of isoquercetin, astragalin, and crypto-chlorogenic acid were calculated from peak area using linear regression from the calibration graph.

### 2.6. Statistical Analysis

 All results were expressed as means ± standard deviations of three replicated determinations by SPSS for Windows 16.0.

## 3. Results and Discussion

**Figure 1:**
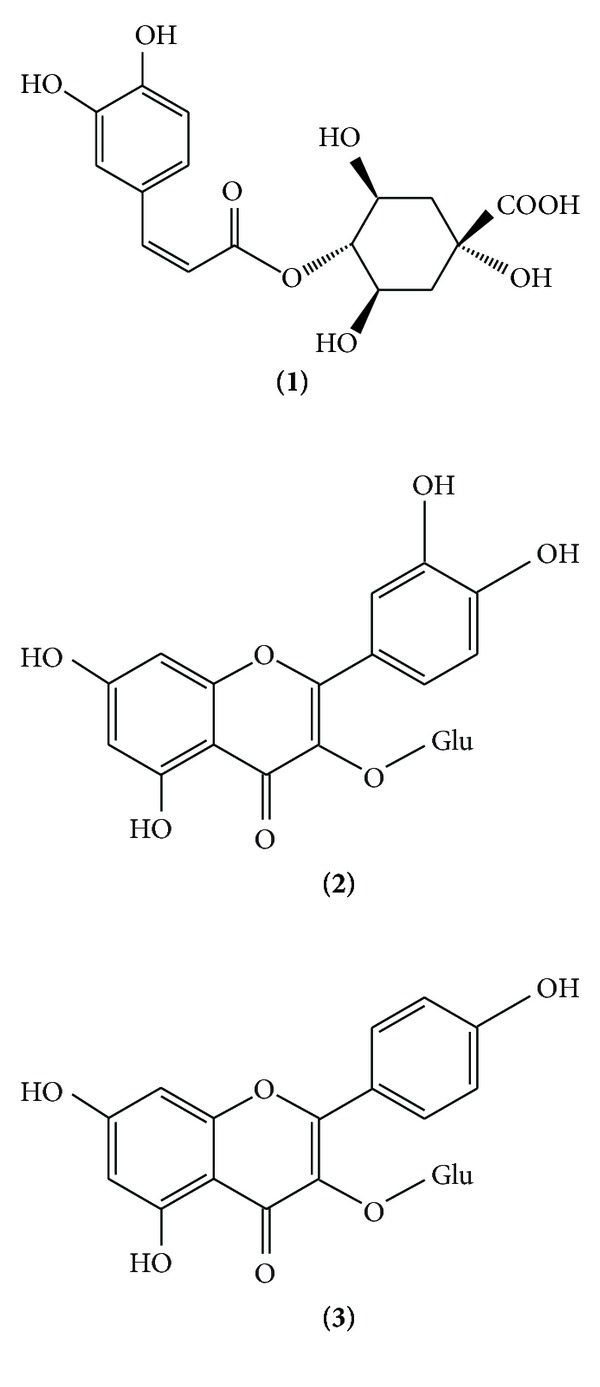
Chemical structures of crypto-chlorogenic acid **(1)**, isoquercetin **(2)** and astragalin **(3)**.

**Figure 2:**
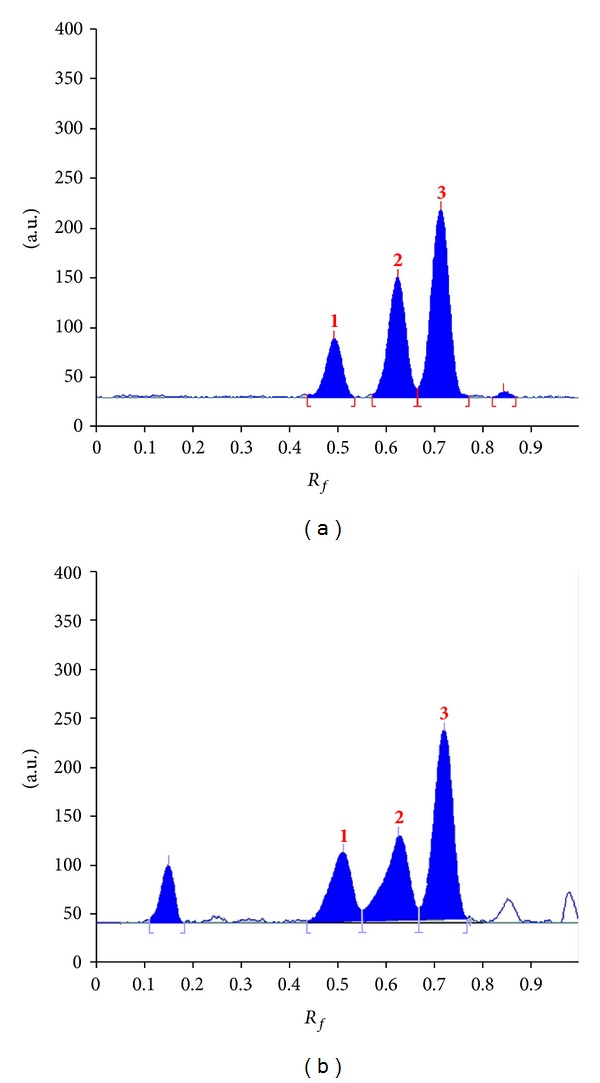
TLC densitograms of standard compounds (a) and the extract from the leaves of *M. oleifera* from Chiang Mai province (b); **1** = crypto-chlorogenic acid, **2** = isoquercetin, and **3** = astragalin.

**Figure 3:**
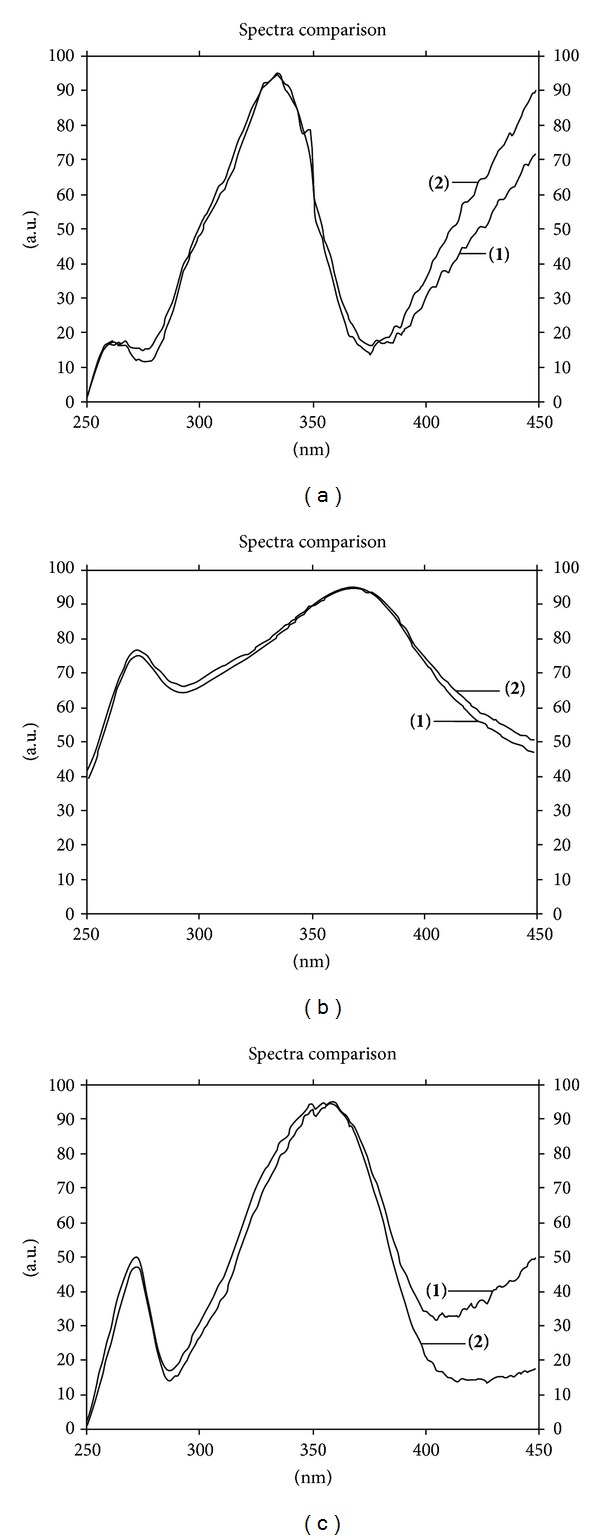
Overlay UV absorption spectra of standard compounds in the extract of *M. oleifera* leaves collected from Chiang Mai province; crypto-chlorogenic acid (a), isoquercetin (b), and astragalin (c); spectra illustrating peak purities of reference standard (**1**) and sample (**2**).

*M. oleifera* leaf extract is composed of complex phenolics and flavonoids [1, 19]. These substances were reported to be biologically active compounds exhibiting several activities such as antidiabetes, antioxidation, and antihyperlipidemia [5–7]. Crypto-chlorogenic acid, isoquercetin, and astragalin (Figure 1) are major components in the leaf extracts of *M. oleifera* [8], and these compounds were employed as markers for TLC-densitometric analysis in the present study. For the chromatographic conditions, several solvents and their proportions of mobile phase were varied to optimize the chromatographic separation. Ethyl acetate : formic acid : acetic acid : water at 34 : 3.5 : 1.5 : 7, v/v/v/v demonstrated the best separation of crypto-chlorogenic acid, isoquercetin, and astragalin with *R*_*f*_ values of 0.51, 0.62, and 0.72, respectively. Densitograms of these three standards and other constituents in the leaf extracts of *M. oleifera* are shown in Figure 2. The identities of the chromatogram bands of these constituents in the sample were confirmed by overlaying their ultraviolet (UV) absorption spectra with those of each standard using the TLC 3 scanner (Figure 3). The method was validated for its linearity, precision, accuracy, LOD, LOQ, and robustness. The linear calibration graphs for crypto-chlorogenic acid, isoquercetin, and astragalin were within the concentration ranges of 103–505, 105–510, and 100–500 ng/spot, with correlation coefficients (*r*) of 0.9961, 0.9975, and 0.9968, respectively (Table 1). The interday and intraday precisions of crypto-chlorogenic acid, isoquercetin, and astragalin were illustrated in Table 2. The results demonstrated acceptable precision of the method, with RSD less than 5% (Table 2). The average recoveries at three different levels of crypto-chlorogenic acid, isoquercetin, and astragalin were 98.73, 98.28, and 99.65%, respectively (Table 3) confirming the accuracy of the method. LOD was found between 10.84 and 14.68 ng/spot and LOQ was in the range of 36.13–48.93 ng/spot (Table 1). For the robustness test, the standard deviation of peak areas was calculated for each parameter and the RSD was found to be less than 3% for all variations (Table 4). The change of mobile phase composition was the crucial deviation of the method while the others were less significant.

The validated TLC-densitometric method was used to determine the contents of crypto-chlorogenic acid, isoquercetin, and astragalin in 12 extracts of *M. oleifera* leaves collected from different locations in Thailand. The amounts of crypto-chlorogenic acid, isoquercetin, and astragalin showed a variation from nondetectable to detectable quantities and provided the averages of 0.0473 ± 0.0236, 0.0427 ± 0.0192, and 0.0534 ± 0.0440% dry weight, respectively (Table 5). The highest contents of these major active components were found in the sample from Chiang Mai province, where the climate is cool, while the lowest contents were in Sa Kaeo province in the warm eastern part of Thailand (Table 5). These findings confirmed the previous report of Iqbal and Bhanger that *M. oleifera* cultivated in the cold place of Pakistan contained the highest contents of total phenolic and total flavonoid compounds [24]. The developed TLC-densitometric method was accurate and precise for the qualitative and quantitative determination of crypto-chlorogenic acid, isoquercetin, and astragalin in *M. oleifera* leaves and was advantageous due to its simplicity and economy. It could be used as an alternative method for routine quantitative analysis of major compounds in the *M. oleifera* leaf extracts. These data will be also useful as guidance for standardization of *M. oleifera* leaf raw materials and for finding good sources of this plant in Thailand. Moreover, the method can be used for quality control of raw materials, extracts, and finished products containing these phenolics and flavonoids.

## 4. Conclusion

The proposed TLC-densitometric method was developed and validated for the simultaneous quantitative analysis of three major active components: crypto-chlorogenic acid, isoquercetin, and astragalin in the extracts of *M. oleifera* leaves collected from 12 different locations in Thailand. The method was simple, accurate, precise and could simultaneously analyze numerous samples. These results will be valuable for further standardization of *M. oleifera *leaves and their extracts, and for indicating good sources of the leaf raw materials of this plant in Thailand. Furthermore, the method could be utilized for quality control of raw materials, extracts, and finished products containing these compounds in commercial nutraceutical and pharmaceutical products.

## Figures and Tables

**Table 1:** Method validation parameters for the quantitation of crypto-chlorogenic acid, isoquercetin, and astragalin by the proposed TLC-densitometric method.

Parameter	Results		
	Crypto-chlorogenic acid	Isoquercetin	Astragalin
Linear range (ng/spot)	103–505	105–510	100–500
Calibration equation	*y* = −2589.922 + 15.123*x*	*y* = −884.153 + 16.772*x*	*y* = 511.081 + 20.486*x*
Correlation coefficient (*r*)	0.9961	0.9975	0.9968
LOQ (ng)	48.93	45.99	36.13
LOD (ng)	14.68	13.80	10.84

*x* is the amount of each standard in ng and *y* is the peak area at 340 nm.

**Table 2:** Intraday and interday precisions of crypto-chlorogenic acid, isoquercetin, and astragalin.

Compounds	Concentration (ng/spot)	Intraday precision* (%)	Interday precision* (%)
Crypto-chlorogenic acid	200	3.17	4.34
	300	2.20	1.13
	400	4.00	3.59

Isoquercetin	200	3.47	4.09
	300	2.43	4.68
	400	4.47	2.47

Astragalin	200	1.56	1.99
	300	1.95	1.56
	400	2.24	2.16

*Present as percent RSD (*n* = 3).

**Table 3:** Recovery studies of crypto-chlorogenic acid, isoquercetin, and astragalin.

Compound	Serial number	Theoretical value (ng)	Amount found* (ng)	Recovery*(%)
Crypto-chlorogenic acid	1	383.47	370.52 ± 8.60	98.67 ± 1.17
	2	393.06	392.69 ± 7.15	99.75 ± 2.75
	3	403.38	402.07 ± 7.89	97.79 ± 3.23

Average				98.73

Isoquercetin	1	325.82	317.49 ± 5.51	98.73 ± 1.74
	2	335.11	328.92 ± 3.92	97.68 ± 1.65
	3	343.64	341.02 ± 3.00	98.43 ± 0.89

Average				98.28

Astragalin	1	287.89	288.21 ± 4.59	100.04 ± 0.90
	2	298.45	298.68 ± 2.74	99.60 ± 1.16
	3	279.59	276.14 ± 6.82	99.32 ± 2.92

Average				99.65

*Expressed as mean ± SD (*n* = 3).

**Table 4:** Robustness studies of crypto-chlorogenic acid, isoquercetin, and astragalin.

Parameter	RSD (%)*		
	Crypto-chlorogenic acid	Isoquercetin	Astragalin
Mobile phase composition ratio	2.99	2.15	1.20
Time from spotting to chromatography	1.59	1.65	0.97
Time from chromatography to scanning	1.16	0.89	0.79
The length of the chromatogram	1.81	1.05	1.21
Presaturation period	2.53	0.66	1.04

*Value from six determinations.

**Table 5:** Contents of crypto-chlorogenic acid, isoquercetin, and astragalin in dried powder of *M. oleifera* leaves collected from various regions of Thailand.

Location (region)	Contents of major compounds (% dry weight)		
	Crypto-chlorogenic acid	Isoquercetin	Astragalin
**Chiang Mai (Northern)**	**0.1021 ± 0.0006**	**0.0733 ± 0.0023**	**0.1604 ± 0.0042**
Lampang (Northern)	0.0693 ± 0.0004	0.0393 ± 0.0017	0.0430 ± 0.0008
Surin (Northeastern)	0.0549 ± 0.0035	0.0638 ± 0.0007	0.0370 ± 0.0004
Udonthani (Northeastern)	0.0411 ± 0.0049	0.0350 ± 0.0057	0.1264 ± 0.0049
Sa Kaeo (Eastern)	ND	ND	0.0192 ± 0.0036
Chonburi (Eastern)	0.0446 ± 0.0003	0.0707 ± 0.0005	0.0287 ± 0.0002
Ang Thong (Central)	0.0602 ± 0.0006	0.0502 ± 0.0044	0.0440 ± 0.0012
Saraburi (Central)	0.0402 ± 0.0011	0.0293 ± 0.0010	0.0219 ± 0.0014
Kanchanaburi (Western)	0.0315 ± 0.0009	0.0230 ± 0.0003	0.0548 ± 0.0010
Phetchaburi (Western)	0.0262 ± 0.0006	0.0198 ± 0.0011	0.0460 ± 0.0004
Phang Nga (Southern)	0.0178 ± 0.0028	0.0258 ± 0.0004	0.0240 ± 0.0024
Phuket (Southern)	0.0327 ± 0.0008	0.0391 ± 0.0021	0.0348 ± 0.0022

Average	0.0473 ± 0.0236	0.0427 ± 0.0192	0.0534 ± 0.0440

*Expressed as mean ± SD (*n* = 3), ND = nondetectable.
